# Delivering injury prevention strategies in police force recruit training: identification of organisational implementation responsibilities

**DOI:** 10.1136/bmjsem-2026-003331

**Published:** 2026-08-04

**Authors:** Myles C Murphy, Dana Hince, Garth Allen, Caroline Bolling, Simon Breheny, Andrea Bruder, Jonathan Hodgson, Joanne Kemp, Debra Langridge, James R McKee, Sophia Nimphius, Vanessa R Sutton, Bruna Tessarin, Evert Verhagen, Andrea B Mosler

**Affiliations:** 1 Centre for Injury Prevention and Pain, Institute for Health Research, The University of Notre Dame Australia, Fremantle, Western Australia, Australia; 2 School of Allied Health, University of Western Australia, Nedlands, Western Australia, Australia; 3 Academy, Western Australia Police Force, Joondalup, Western Australia, Australia; 4 Amsterdam Collaboration on Health and Safety in Sports, Department of Orthopedic Surgery and Sports Medicine, Amsterdam UMC Locatie AMC, Amsterdam, Netherlands; 5 Western Australia Police Force, Perth, Western Australia, Australia; 6 La Trobe Sport and Exercise Medicine Research Centre, La Trobe University, Melbourne, Victoria, Australia; 7 Physiotherapy, Podiatry, Prosthetics and Orthotics, School of Allied Health, Human Services and Sport, La Trobe University, Melbourne, Victoria, Australia; 8 Nutrition and Health Innovation Research Institute, Edith Cowan University, Joondalup, Western Australia, Australia; 9 Western Australian Health Translation Network, The University of Western Australia, Perth, Western Australia, Australia; 10 Edith Cowan University, Joondalup, Western Australia, Australia; 11 Medical and Anti-Doping, Football Division, Union of European Football Association, Nyon, Switzerland

**Keywords:** Implementation, Injury, Prevention

## Abstract

**Objectives:**

To reduce injury burden among police recruits we must identify who is responsible for implementing prevention strategies. Using a novel methodology, we aimed to examine the perspectives of police force recruits and staff to determine who they perceived to be responsible to implement important injury prevention strategies.

**Methods:**

We performed a cross-sectional study co-designed by our broader consumer, industry and research advisory groups. Five data collection sessions were conducted, involving a total of nine separate groups; analyses were performed by group rather than at the individual level. Groups were clustered by organisational role: management (n=2 groups), training delivery (n=3 groups) and recruits (n=4 groups). Participants allocated 57 previously identified important injury prevention strategies to the group(s) ((1) recruits, (2) training delivery, (3) management) responsible for implementing each strategy.

**Results:**

Participants identified almost three-quarters of prevention strategies required shared responsibility among recruits, training delivery staff and/or management. No strategies were considered the sole responsibility of recruits.

**Conclusion:**

Our research demonstrated that while recruits must take ownership and responsibility for some aspects of their training no prevention priority areas are the sole responsibility of recruits. Thus, recruit injury prevention must be implemented by recruits, training delivery staff and management.

**WHAT IS ALREADY KNOWN ON THIS TOPIC**

- Important and feasible strategies to reduce the burden of injury in police force recruits have been identified.

**WHAT THIS STUDY ADDS**

- Shared responsibility between management staff, training delivery staff and recruits is required to improve the likelihood of successful implementation of injury prevention strategies.

**HOW THIS STUDY MIGHT AFFECT RESEARCH, PRACTICE OR POLICY**

- This study presents a new methodology to assist in the implementation of injury prevention strategies by allocating responsibility of proposed intervention strategies to specific groups.

## Introduction

 Police force recruits experience high musculoskeletal injury burden during their recruit training, with most injuries affecting the lower limbs.^1–3^ A body of research has identified risk factors associated with these injuries (eg, older age, lower cardiorespiratory fitness);^4^ however, few clinical trials have examined the effectiveness of prevention interventions (eg, exercise-based interventions targeting modifiable risk factors) in police force recruits.^5^ Further, while modifiable risk factors may identify ‘what’ intervention might be needed,^6^ there is limited data to inform ‘who’ should implement prevention strategies.

Exercise-based training interventions have been successfully implemented in sporting environments,^7^ reducing the incidence of musculoskeletal injuries.^8–10^ However, contextual factors in police force recruit training programmes, such as recruitment diversity (eg, increasing heterogeneity with more older recruits), occupational requirements (eg, competing time allocation for classroom and operational activities) and organisational policies (eg, physical entrance standards),^11 12^ may limit the simple adaptation of prevention programmes developed for athletes into the tactical training environment. We conducted a concept-mapping study including representation from the police force, healthcare providers and researchers.^13^ We identified eight injury prevention priority areas, consisting of 57 important strategies to prevent injury during recruit training programmes (online supplemental appendix A).^13^ Now we have identified ‘what’ can be done to reduce the injury burden, we must identify ‘who’ is responsible for implementing these strategies to mitigate barriers and leverage facilitators.^14^ We aimed to examine the perspectives of police force recruits and staff to determine who they perceived to be responsible to implement the 57 prevention strategies identified in our concept-mapping study.

## Methods

### Study design

We performed a cross-sectional study, co-designed by our broader consumer, industry and research advisory groups,^15 16^ to assist in planning intervention implementation.^17^

### Setting

Five data collection sessions were conducted in September 2025, with four held in-person at the Western Australia (WA) Police Force Academy site and one delivered online via Microsoft Teams to accommodate those unable to attend in-person.

### Participants

All current WA Police Force Academy recruits and staff from a range of divisions (eg, Physical Performance Unit, Training Management Support, Occupational Health and Safety) were invited to participate via email by a member of the WA Police Force Academy. Participant demographics (gender, self-reported ethnicity, education level, number of self-reported injury regions in the past 12 months) were recorded via Qualtrics survey software (Provo, Utah, USA).

### Data sources and variables

Five data collection sessions were conducted, involving a total of nine separate groups; analyses were performed by group, rather than at the individual level. Groups were clustered by organisational role: management (n=2 groups), training delivery (n=3 groups) and recruits (n=4 groups). Our research identified 57 prevention strategies deemed ‘most’ important across eight priority areas (online supplemental appendix A).^13^ Each of the nine groups was instructed to allocate the 57 injury prevention strategies to the group(s) responsible for implementing each prevention strategy. Participants were provided a large magnetic whiteboard with a Venn diagram and asked to allocate each prevention strategy (printed on a magnet) to the appropriate zone on the Venn diagram (three responsibility groups: recruit, training delivery, management or any combination). For our online group, this exercise was performed with one investigator (MCM) moving strategies onto the whiteboard on behalf of participants. Participants were required to collectively agree, via discussion within their group, on where to place each strategy on the Venn diagram. Participants resolved disputes through further discussion until dispute resolution.

### Statistical analysis

Participant demographics are presented descriptively, as appropriate. The data from all responsibility allocation boards was converted to an MS Excel spreadsheet, and the mode allocation of each prevention strategy was calculated and presented visually using a Venn diagram. To assess agreement for the prevention strategy within each priority area, a binary variable (coded ‘Yes, responsible’ or ‘Not responsible’) for each strategy, for each responsibility group, based on the responses from each participant cluster was constructed for: (1) recruit, (2) training delivery and (3) management. Fleiss’ kappa (indicating agreement irrespective of response direction (yes/no)) was calculated for the eight priority areas. A separate analysis was conducted for whether responsibility for implementation lies with: (1) recruit, (2) training delivery and (3) management. When the kappa score was positive, a p value of <0.05 was used to indicate agreement above that expected by chance.

## Results

### Demographics

23 members of the WA Police Force Academy, including management staff (n=8), training delivery staff (n=6) and recruits (n=9), were purposively recruited. Median (IQR) ages were 25.0 (8.0) years for recruits, 34.5 (11.0) years for training delivery staff and 43.0 (8.0) years for management staff. Further demographic information is included in online supplemental appendix B.

### Responsibility allocation

**Figure 1:**
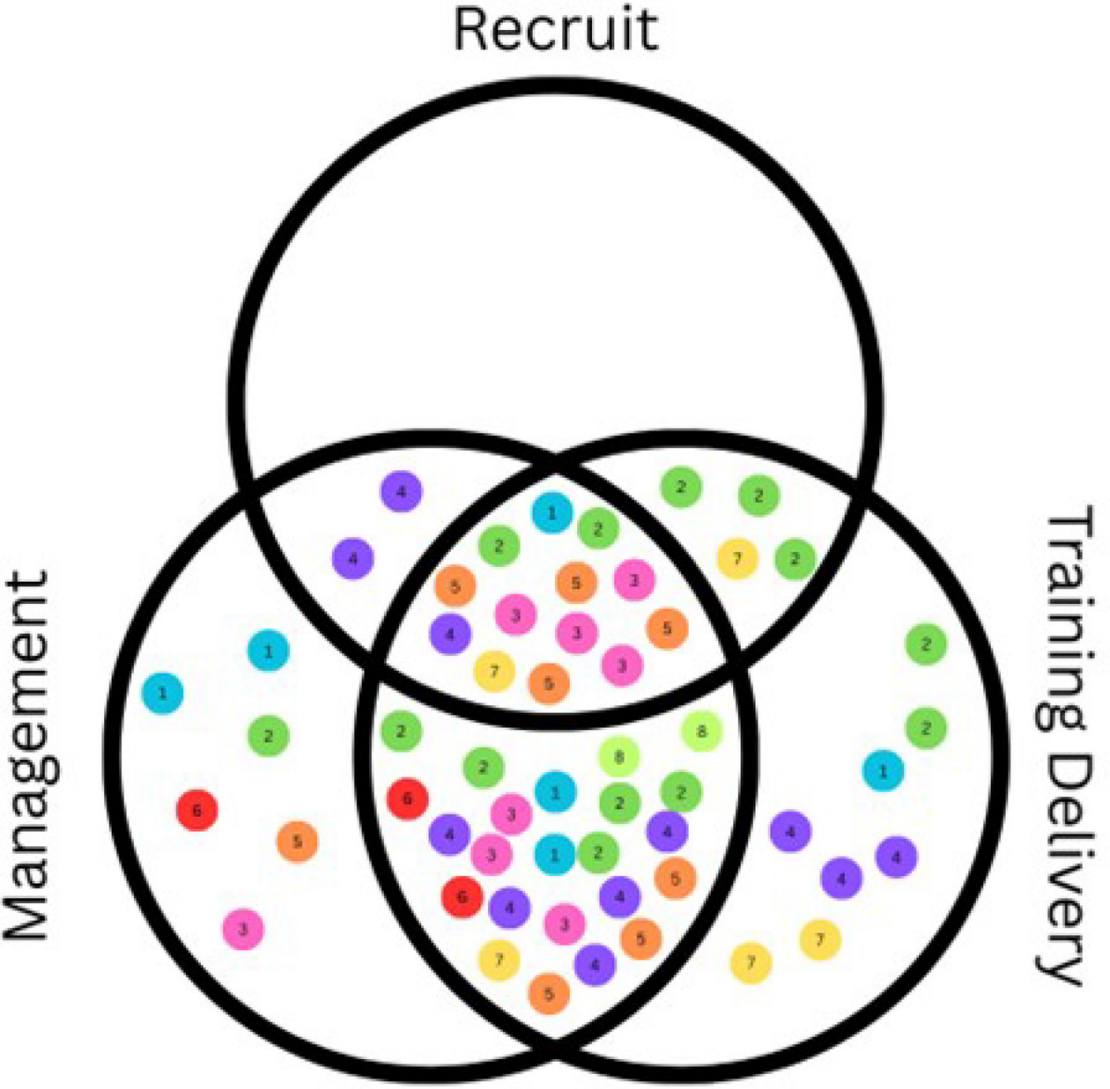
Overall injury prevention strategy implementation responsibility map with numbers and colours representing the priority area reported in table 1: (1) Clearly communicate physical training programme expectations and requirements; (2) prepare for, monitor and manage physical training load; (3) provide best practice injury identification, prevention and management; (4) educate recruits, staff and other stakeholders involved in academy training delivery; (5) provide a supportive training environment that promotes health, well-being and injury reporting; (6) have experienced staff deliver training and use appropriate equipment; (7) deliver a comprehensive and holistic physical training programme; and (8) have appropriate physical entry standards and requirements.

The overall agreement (yes/no) on responsibility allocation across priority areas is presented (figure 1), with each individual strategy allocation presented in online supplemental appendix C. Three-quarters (75%, 43/57) of prevention strategies were allocated a shared responsibility among two or more organisational roles. The statistical agreement is reported in table 1. Priority area 1: ‘Clearly communicate physical training program expectations and requirements’ was agreed to be the responsibility of management (p<0.001) and not of recruits (p=0.002). Priority area 2: ‘Prepare for, monitor and manage physical training load’ was agreed to be the shared responsibility of management (p=0.043) and training delivery staff (p=0.028). Priority area 3: ‘Provide best practice injury identification, prevention and management’ was agreed to be the responsibility of training delivery staff (p<0.001) and not recruits (p<0.001). Priority area 4: ‘Educate recruits, staff and other stakeholders involved in academy training delivery’ was agreed to be the shared responsibility of management (p<0.001) and training delivery staff (p=0.001) but not recruits (p=0.001). Priority area 5: ‘Provide a supportive training environment that promotes health, wellbeing and injury reporting’ was agreed to be the responsibility of training delivery staff (p=0.006) but not recruits (p<0.001). Priority area 6: ‘Have experienced staff deliver training and use appropriate equipment’ was agreed to be the responsibility of training delivery staff (p=0.042). There was no agreement in responsibility allocation for Priority area 7: ‘Deliver a comprehensive and holistic physical training program’. Priority area 8: ‘Have appropriate physical entry standards and requirements’ was deemed not to be the responsibility of recruits by all groups (ie, raters).

**Table 1:** Fleiss’ kappa calculated for nine groups (ie, raters) across prevention strategies included in each of the eight injury prevention priority areas

Injury prevention priority area (no. prevention strategies)	Statistic	Police force group responsible for supporting implementation of the prevention strategy (n=9 groups)		
		Management	Training delivery	Recruits
Clearly communicate physical training programme expectations and requirements (n=6)	Yes^1^ (%)	79.6	66.7	29.6
	Kappa	0.54	0.10	0.20
	Z	7.99	1.53	2.95
	p	**<0.001**	0.063	**0.002**
Prepare for, monitor and manage physical training load (n=13)	Yes^1^ (%)	61.5	85.5	48.7
	Kappa	0.08	0.09	0.04
	Z	1.71	1.91	0.91
	p	**0.043**	**0.028**	0.181
Provide best practice injury identification, prevention and management (n=9)	Yes^1^ (%)	88.9	85.2	44.4
	Kappa	−0.03	0.29	0.49
	Z	−0.56	5.23	8.77
	p	0.713	**<0.001**	**<0.001**
Educate recruits, staff and other stakeholders involved in academy training delivery (n=11)	Yes^1^ (%)	73.7	80.8	39.4
	Kappa	0.39	0.15	0.15
	Z	7.70	3.05	3.06
	p	**<0.001**	**0.001**	**0.001**
Provide a supportive training environment that promotes health, well-being and injury reporting (n=8)	Yes^1^ (%)	80.6	75.0	44.4
	Kappa	0.07	0.15	0.35
	Z	1.17	2.51	5.99
	p	0.121	**0.006**	**<0.001**
Have experienced staff deliver training and use appropriate equipment (n=3)	Yes^1^ (%)	77.8	66.7	22.2
	Kappa	−0.07	0.17	−0.13
	Z	−0.74	1.73	−1.30
	p	0.771	**0.042**	0.903
Deliver a comprehensive and holistic physical training programme (n=5)	Yes^1^ (%)	37.8	86.7	55.6
	Kappa	−0.06	−0.01	0.08
	Z	−0.85	−0.13	1.04
	p	0.803	0.551	0.149
Have appropriate physical entry standards and requirements (n=2)	Yes^1^ (%)	77.8	72.2	0.0
	Kappa	−0.04	−0.11	NC
	Z	−0.38	−0.91	NC
	p	0.648	0.820	NC

Z statistic and p value test the hypothesis that kappa >0 (ie, agreement is above chance level). Significance was accepted when p<0.05 and marked in bold. Negative kappa indicates agreement below chance level. NC because all groups (ie, raters) agreed both items in this priority area were not the responsibility of recruits (ie, all chose not responsible). Interpretation of the kappa statistic was quantified with the mean percentage of groups who responded yes to all strategies included in each priority area because ‘agreement’ indicates the same response, not the direction of response, in the two-response case. 1Percentage of the nine groups who responded ‘Yes, this is the responsibility of X’ averaged (mean) across all prevention strategies in the given priority area, where >50% indicated agreement for yes responsible, whereas <50% indicated agreement for not responsible. Priority area colours correspond to the colours in figure 1.

NC, not calculable.

### Patient and public involvement

Our research was co-designed and conducted with consumer and industry advisory groups. The results were presented to police force recruits and officers during an advisory group meeting.^16^ The consumer advisory group felt strongly that despite these results, recruits need to take greater responsibility and not only rely on the training programme to provide everything they need to be healthy and injury-free. Therefore, while not an identified prevention intervention ‘priority area’ our consumers highlighted the importance of recruits being responsible for maintaining their overall health.

## Discussion

Police force recruits, recruit training delivery staff and recruit training management staff were engaged to allocate implementation responsibility to 57 important injury prevention strategies for police force recruits.^13^ We found that shared responsibility between management staff, training delivery staff and recruits is required to improve the likelihood of successful implementation of an injury prevention intervention.

Our study findings revealed that 75% (n=43) of individual injury prevention strategies were allocated a shared responsibility between either management staff, training delivery staff or recruits. However, recruits were not assigned isolated responsibility for any prevention strategy, and explicitly not responsible for four of the eight identified injury prevention priority areas (priority areas 1, 3, 4 and 5). This contrasts with previous injury prevention research, which has traditionally focussed on the end user being responsible for delivering or performing the intervention. Thus, while interventions may be for recruit benefit, they were not considered responsible for implementing any of the injury prevention strategies alone. This highlights the potential importance of ensuring those designing prevention interventions include broad participant representation with the relevant lived experiences to co-design interventions and implementation strategies.

Our findings also suggest that the effectiveness of previous interventions may be limited by targeting only one group involved in the delivery of injury prevention interventions. Instead, our results suggest we must involve the staff delivering and managing recruit training from ideation through to translation, which will assist in successful implementation while reducing unnecessary responsibility allocation. However, our consumer advisory group highlighted that while responsibility for injury prevention strategy implementation may not be the sole responsibility of recruits, this should not diminish an individual recruits’ general accountability for their own health, which was viewed as a general requirement of policing, and not a specific ‘strategy’.

### Limitations

While we included 23 participants in this study, we analysed agreement between groups (n=9), thereby reducing our effective sample size. However, as all injury prevention strategies were discussed within each group, we felt it was inappropriate to assume the group strategy allocation for all participants would be the same as the group decision without discussion and consensus. Finally, we did not explore subgroups within recruits, training delivery, and management.

## Conclusion

Our study provides clear recommendations for the implementation of an injury prevention intervention for a police force context, addressing an implementation gap. We demonstrated that almost three-quarters of individual police recruit injury prevention strategies were considered shared responsibility between either recruits, training delivery staff and/or management. Our research demonstrated that whilst recruits must take ownership and responsibility for some aspects of their training (eg, adherence, effort, reporting injuries, self-care) no prevention strategies are the sole responsibility of recruits. Thus, recruit injury prevention must be implemented by recruits, training delivery staff and management.

## Supplementary material

10.1136/bmjsem-2026-003331 online supplemental file 1
