# Supplementary material for: Delivering injury prevention strategies in police force recruit training: identification of organisational implementation responsibilities
Source: BMJ Open Sport Exerc Med. 2026 Aug 4;12(3):e003331. doi: 10.1136/bmjsem-2026-003331 (PMC13448528; doi:10.1136/bmjsem-2026-003331)
Supplement: Supplementary file 1 [file bmjsem-12-3-s001.docx]

**Appendix A. The prevention strategies ranked as above the grand mean for importance, mapped to priority areas, from Murphy et al. 2026.**

| **Priority Area** | **Prevention Strategy** |
| --- | --- |
| 1: Clearly communicate physical training program expectations and requirements | communicate physical fitness expectations within recruitment material |
|  | develop and provide pre-habilitation guidelines/recommendations/programs to recruits before starting the recruit training program |
|  | ensure recruit training program staff have early knowledge of recruits' physical standards |
|  | provide guidance to recruits on appropriate ways to monitor and increase training volume over time |
|  | raise awareness that minimum fitness standards are not high enough to prevent injury or meet the physical capacity required to complete the program, and set the bar high for recruits to rise to, rather than allowing recruits to only train to minimum standards |
|  | undertake a generalised injury prevention program for everyone |
| 2: Prepare for, monitor and manage physical training load | create optimal baseline targets for strength/endurance |
|  | ensure consistent physical training schedules to minimise large fluctuations in training load (e.g. four sessions one week, none the next) |
|  | gradually increase body armour load, instead of adding it all at once |
|  | maintain standards of fitness across the entire duration of recruit training |
|  | monitor training load and volume within the program |
|  | perform baseline screening for previous medical and injury history to inform recruit training |
|  | perform baseline strength, power and conditioning screening to inform training |
|  | provide adequate time to recover physically and mentally in between drills that stress the same body area |
|  | provide flexibility within the sessions based on physical fatigue / injuries / mental fatigue |
|  | review recruits' training and exercise history and adjust loading/requirements as appropriate |
|  | treat fitness requirements the same as critical skills training. |
|  | undertake an annual review of training programs and testing methods |
|  | undertake physical tests that appropriately reflect workforce requirements (e.g. running in boots, carrying extra weight) |
| 3: Provide best practice injury identification, prevention and management | employ a full-time physiotherapist based at the recruit training program |
|  | employ clear goal setting post injury and monitor progress. |
|  | ensure physical trainers are supervised by and have access to appropriately qualified/accredited support staff (e.g. physiotherapists, exercise physiologists, occupational physicians) |
|  | have a system in place to support recruits throughout the injury recovery process (e.g. communication pathways, opportunity to modify training content |
|  | have appropriate pathways and management for recruits at high-risk of ongoing injury burden or when recovery is not progressing as planned |
|  | monitor and manage the mental health of recruits throughout recruit training |
|  | provide a harmonious, supportive, and inclusive social environment to ensure recruits feel valued and supported within the training program |
|  | support injury return by joint decision-making and shared responsibility between health care professionals, the recruit and police instructors |
|  | use a range of experienced and well-qualified health professionals with experience in musculoskeletal injury management |
| 4: Educate recruits, staff and other stakeholders involved in academy training delivery | complete mandatory education on how to optimise their performance and technique before starting the recruit training program |
|  | complete mandatory education on how to safely address and reduce the impact of injuries before starting the recruit training program |
|  | educate about and facilitate access to holistic recovery strategies (psychological, social, mental, and physical) |
|  | educate all stakeholders involved about injury prevention programs, including officers, recruits and other stakeholders of relevance |
|  | educate recruits about common soft-tissue injuries, and how/why they happen |
|  | educate recruits on recovery, including sleep hygiene |
|  | educate recruits, physical trainers and law enforcement staff on the importance of early injury recognition and intervention |
|  | include nutrition education (e.g. daily nutrition needs, meal preparation, injury recovery, reduce inflammation etc.) |
|  | provide education about the expected response to physical training (e.g. muscle soreness vs. muscle injury) |
|  | provide education on injury reporting (what, who, when) |
|  | understand the patterns, cause and nature of the injury/ies |
| 5: Provide a supportive training environment that promotes health, wellbeing and injury reporting | build and maintain a culture that enables recruits to report physical complaints early (e.g. not seeing injuries as being "weak" or "soft") |
|  | ensure adequate training of the physical training instructors |
|  | ensure good relationships between recruits, physical trainers, exercise physiologists, physiotherapists and occupational physicians engaged within the recruit training program |
|  | ensure that sworn police force officers maintain physical fitness to show recruits that it is crucial to being a police officer |
|  | monitor and address factors, such as fatigue, distress, wellbeing, are addressed, so that recruits can be their best self and fully attentive |
|  | provide qualified and experienced strength and conditioning coaches to supervise, program, and deliver physical training sessions |
|  | provide recruit training program staff with access to resources and expertise on mental health, psychology, and wellbeing to better support their recruits |
|  | support recovery post-strenuous activities (e.g. nutrition, rest, sleep, ice baths, massage etc.) |
| 6: Have experienced staff deliver training and use appropriate equipment | complete all pre-medicals and baseline testing by recruit training program staff |
|  | identify specific equipment that may impact injury risk or burden (e.g. obstacle course, weight of vest or belt) |
|  | provide high quality personal protective equipment for tactical, physical, and combat training |
| 7: Deliver a comprehensive and holistic physical training program | design training programs that build functional strength and fitness |
|  | focus on strength exercises |
|  | provide a strength and conditioning program before entering the recruit training program for those identified as not up to physical standard |
|  | use training which reflects required functional activities of law enforcement officers |
|  | warm-up appropriately (including dynamic movements) for all training content |
| 8: Have appropriate physical entry standards and requirements | complete recruits' final physical assessment closer to when they enter the recruit training program |
|  | increase the physical entrance test standard (e.g. higher beep test level) |

**Appendix B. Participant demographic information**

| **Domain** | **Variable** | **Recruit (n=9)** | **Training Delivery (n=6)** | **Management (n=8)** |
| --- | --- | --- | --- | --- |
| Gender | Man or male | 5 | 3 | 4 |
|  | Woman or female | 4 | 3 | 4 |
| Self-reported ethnicity | Australian | 4 | 2 | 4 |
|  | Caucasian | 3 | 0 | 2 |
|  | European | 1 | 3 | 2 |
|  | South African | 0 | 1 | 0 |
|  | Māori | 1 | 0 | 0 |
| Education Level | Less than high school degree | 2 | 0 | 1 |
|  | High school graduate (high school diploma or equivalent including GED) | 5 | 1 | 0 |
|  | Bachelor's degree | 2 | 2 | 6 |
|  | Master's degree | 0 | 3 | 1 |
| Number of self-reported injury regions in the past 12 months | 0 injury regions | 4 | 3 | 2 |
|  | 1 injury region | 4 | 2 | 1 |
|  | 2 injury regions | 1 | 0 | 2 |
|  | 3 injury regions | 0 | 0 | 0 |
|  | 4 injury regions | 0 | 0 | 0 |
|  | 5 injury regions | 0 | 1 | 1 |
|  | 6 injury regions | 0 | 0 | 0 |
|  | 7 injury regions | 0 | 0 | 0 |
|  | 8 injury regions | 0 | 0 | 0 |
|  | 9 injury regions | 0 | 0 | 2 |

**Appendix C. Allocation of responsibility by prevention strategy.**

| **Domain** | **Prevention Strategy** | **Management** | **Training delivery** | **Recruit** |
| --- | --- | --- | --- | --- |
| Clearly communicate physical training program expectations and requirements | Communicate physical fitness expectations within recruitment material | 1 | 0 | 0 |
|  | Develop and provide pre-habilitation guidelines/recommendations/programs to recruits before starting the recruit training program | 1 | 1 | 0 |
|  | Ensure recruit training program staff have early knowledge of recruits' physical standards | 1 | 0 | 0 |
|  | Provide guidance to recruits on appropriate ways to monitor and increase training volume over time | 0 | 1 | 0 |
|  | Raise awareness that minimum fitness standards are not high enough to prevent injury or meet the physical capacity required to complete the program, and set the bar high for recruits to rise to, rather than allowing recruits to only train to minimum standards | 1 | 1 | 0 |
|  | Undertake a generalised injury prevention program for everyone | 1 | 1 | 1 |
| Prepare for, monitor and manage physical training load | Create optimal baseline targets for strength/endurance | 1 | 1 | 0 |
|  | Ensure consistent physical training schedules to minimise large fluctuations in training load (e.g. Four sessions one week, none the next) | 1 | 1 | 0 |
|  | Gradually increase body armour load, instead of adding it all at once | 0 | 1 | 0 |
|  | Maintain standards of fitness across the entire duration of recruit training | 0 | 1 | 1 |
|  | Monitor training load and volume within the program | 0 | 1 | 1 |
|  | Perform baseline screening for previous medical and injury history to inform recruit training | 1 | 1 | 0 |
|  | Perform baseline strength, power and conditioning screening to inform training | 0 | 1 | 0 |
|  | Provide adequate time to recover physically and mentally in between drills that stress the same body area | 1 | 1 | 1 |
|  | Provide flexibility within the sessions based on physical fatigue / injuries / mental fatigue | 0 | 1 | 1 |
|  | Review recruits' training and exercise history and adjust loading/requirements as appropriate | 1 | 1 | 0 |
|  | Treat fitness requirements the same as critical skills training. | 1 | 1 | 1 |
|  | Undertake an annual review of training programs and testing methods | 1 | 1 | 0 |
|  | Undertake physical tests that appropriately reflect workforce requirements (e.g. Running in boots, carrying extra weight) | 1 | 1 | 1 |
| Provide best practice injury identification, prevention and management | Employ a full-time physiotherapist based at the recruit training program | 1 | 0 | 0 |
|  | Employ clear goal setting post injury and monitor progress. | 1 | 1 | 1 |
|  | Ensure physical trainers are supervised by and have access to appropriately qualified/accredited support staff (e.g. Physiotherapists, exercise physiologists, occupational physicians) | 1 | 1 | 0 |
|  | Have a system in place to support recruits throughout the injury recovery process (e.g. Communication pathways, opportunity to modify training content | 1 | 1 | 0 |
|  | Have appropriate pathways and management for recruits at high-risk of ongoing injury burden or when recovery is not progressing as planned | 1 | 1 | 0 |
|  | Monitor and manage the mental health of recruits throughout recruit training | 1 | 1 | 1 |
|  | Provide a harmonious, supportive, and inclusive social environment to ensure recruits feel valued and supported within the training program | 1 | 1 | 1 |
|  | Support injury return by joint decision-making and shared responsibility between health care professionals, the recruit and police instructors | 1 | 1 | 1 |
|  | Use a range of experienced and well-qualified health professionals with experience in musculoskeletal injury management | 1 | 1 | 0 |
| Educate recruits, staff and other stakeholders involved in academy training delivery | Complete mandatory education on how to optimise their performance and technique before starting the recruit training program | 1 | 0 | 1 |
|  | Complete mandatory education on how to safely address and reduce the impact of injuries before starting the recruit training program | 1 | 0 | 1 |
|  | Educate about and facilitate access to holistic recovery strategies (psychological, social, mental, and physical) | 1 | 1 | 0 |
|  | Educate all stakeholders involved about injury prevention programs, including officers, recruits and other stakeholders of relevance | 1 | 1 | 0 |
|  | Educate recruits about common soft-tissue injuries, and how/why they happen | 0 | 1 | 0 |
|  | Educate recruits on recovery, including sleep hygiene | 1 | 1 | 0 |
|  | Educate recruits, physical trainers and law enforcement staff on the importance of early injury recognition and intervention | 1 | 1 | 0 |
|  | Include nutrition education (e.g. Daily nutrition needs, meal preparation, injury recovery, reduce inflammation etc.) | 0 | 1 | 0 |
|  | Provide education about the expected response to physical training (e.g. Muscle soreness vs. Muscle injury) | 0 | 1 | 0 |
|  | Provide education on injury reporting (what, who, when) | 1 | 1 | 0 |
|  | Understand the patterns, cause and nature of the injury/ies | 1 | 1 | 1 |
| Provide a supportive training environment that promotes health, wellbeing and injury reporting | Build and maintain a culture that enables recruits to report physical complaints early (e.g. Not seeing injuries as being "weak" or "soft") | 1 | 1 | 1 |
|  | Ensure adequate training of the physical training instructors | 1 | 1 | 0 |
|  | Ensure good relationships between recruits, physical trainers, exercise physiologists, physiotherapists and occupational physicians engaged within the recruit training program | 1 | 1 | 1 |
|  | Ensure that sworn police force officers maintain physical fitness to show recruits that it is crucial to being a police officer | 1 | 1 | 0 |
|  | Monitor and address factors, such as fatigue, distress, wellbeing, are addressed, so that recruits can be their best self and fully attentive | 1 | 1 | 1 |
|  | Provide qualified and experienced strength and conditioning coaches to supervise, program, and deliver physical training sessions | 1 | 1 | 0 |
|  | Provide recruit training program staff with access to resources and expertise on mental health, psychology, and wellbeing to better support their recruits | 1 | 0 | 0 |
|  | Support recovery post-strenuous activities (e.g. Nutrition, rest, sleep, ice baths, massage etc.) | 1 | 1 | 1 |
| Have experienced staff deliver training and use appropriate equipment | Complete all pre-medicals and baseline testing by recruit training program staff | 1 | 1 | 0 |
|  | Identify specific equipment that may impact injury risk or burden (e.g. Obstacle course, weight of vest or belt) | 1 | 1 | 0 |
|  | Provide high quality personal protective equipment for tactical, physical, and combat training | 1 | 0 | 0 |
| Deliver a comprehensive and holistic physical training program | Design training programs that build functional strength and fitness | 0 | 1 | 0 |
|  | Focus on strength exercises | 0 | 1 | 1 |
|  | Provide a strength and conditioning program before entering the recruit training program for those identified as not up to physical standard | 1 | 1 | 1 |
|  | Use training which reflects required functional activities of law enforcement officers | 0 | 1 | 0 |
|  | Warm-up appropriately (including dynamic movements) for all training content | 0 | 1 | 1 |
| Have appropriate physical entry standards and requirements | Complete recruits' final physical assessment closer to when they enter the recruit training program | 1 | 1 | 0 |
|  | Increase the physical entrance test standard (e.g. Higher beep test level) | 1 | 1 | 0 |
